# TLGP: a flexible transfer learning algorithm for gene prioritization based on heterogeneous source domain

**DOI:** 10.1186/s12859-021-04190-9

**Published:** 2021-08-25

**Authors:** Yan Wang, Zuheng Xia, Jingjing Deng, Xianghua Xie, Maoguo Gong, Xiaoke Ma

**Affiliations:** 1 School of Computer Science and Technology, Xidian University, South TaiBai Road, Xi’an, China; 2 Department of Library, Xidian University, South TaiBai Road, Xi’an, China; 3 Department of Computer Science, Swansea University, Bay, UK; 4 School of Electronic Engineering, Xidian University, South TaiBai Road, Xi’an, China

**Keywords:** Gene prioritizatio, Transfer learning, Gene co-expression network, Integrative analysis

## Abstract

**Background:**

Gene prioritization (gene ranking) aims to obtain the centrality of genes, which is critical for cancer diagnosis and therapy since keys genes correspond to the biomarkers or targets of drugs. Great efforts have been devoted to the gene ranking problem by exploring the similarity between candidate and known disease-causing genes. However, when the number of disease-causing genes is limited, they are not applicable largely due to the low accuracy. Actually, the number of disease-causing genes for cancers, particularly for these rare cancers, are really limited. Therefore, there is a critical needed to design effective and efficient algorithms for gene ranking with limited prior disease-causing genes.

**Results:**

In this study, we propose a transfer learning based algorithm for gene prioritization (called TLGP) in the cancer (target domain) without disease-causing genes by transferring knowledge from other cancers (source domain). The underlying assumption is that knowledge shared by similar cancers improves the accuracy of gene prioritization. Specifically, TLGP first quantifies the similarity between the target and source domain by calculating the affinity matrix for genes. Then, TLGP automatically learns a fusion network for the target cancer by fusing affinity matrix, pathogenic genes and genomic data of source cancers. Finally, genes in the target cancer are prioritized. The experimental results indicate that the learnt fusion network is more reliable than gene co-expression network, implying that transferring knowledge from other cancers improves the accuracy of network construction. Moreover, TLGP outperforms state-of-the-art approaches in terms of accuracy, improving at least 5%.

**Conclusion:**

The proposed model and method provide an effective and efficient strategy for gene ranking by integrating genomic data from various cancers.

## Background

Genes are basic units of organisms, which execute critical biological processes to maintain the operation of life. And, DNA mutations change the sequences of genes, resulting in variations of gene structure and functions, which originate cancers [1]. Therefore, genes serve as bio-markers for cancer diagnosis and target genes of drugs, which are the foundation of cancer therapy [2, 3]. It is of great significance to identify pathogenic genes for revealing the underlying mechanisms of cancers because it helps biological researchers to handle mountains of public and private omics data to maximize the yield of downstream biological validation.

Pathogenic gene detection corresponds to the gene prioritization problem, which aims to ranking genes according their importance, where important genes are more likely to be pathogenic. Great efforts have been devoted to gene ranking, which can be categorized into two groups, i.e. biological experiment- and computation-based approaches. The methods of the first category validate the functions and structure of genes to select pathogenic genes by employing biological experiments. The advantage of biological experiment-based methods is accurate, whereas the drawback is time- and finance-consuming. To overcome these issues, the computation-based methods provide an alternative for experiment-based methods, which utilize machine learning techniques to predict the possible pathogenic genes by exploiting genomic data of cancers. The underlying assumption for computational based algorithms is that genes with similar structure have similar biological functions and patterns [4–6].

Many algorithms have been developed for gene ranking [7–16], where the difference among them lies on how to define and measure the similarity between the pathogenic and non-pathogenic genes. The most intuitive and straightforward strategy is to calculate the distance between pathogenic and non-pathogenic genes in terms of features [8]. If the candidate gene is very close to pathogenic genes, it is reasonable to consider the candidate gene as pathogenic genes. The key factor behind the similarity strategy is how to construct the features for genes. And, algorithms employ various types of features, for example, PROSPECTR [17] explores sequence-based features. However, feature similarity approaches are criticized for the low accuracy because they only explore the relation between a pair of genes. To solve this problem, many classification algorithms are adopted to predict pathogenic genes, including rule-base decision tree [18] and support vector machine (SVM) [19]. These algorithms significantly outperform the feature similarity strategy since they make use of features of whole genes. To further improve the performance of algorithms, Moreau et al. [20] suggest that it is promising to integrate complex and heterogeneous data to identify the most interesting genes for biological validation from candidates.

Even though the classification-based methods achieve an excellent performance on gene prioritization, they require a large number of positive and negative samples to ensure the reliability of classifiers. When the training set is insufficient, these algorithms are criticized for the low accuracy. Furthermore, they cannot explore the indirect relations among genes. Network is a powerful tool for characterizing and describing the complex systems, which has been successfully applied to social analysis [21–24] and biology [25–32]. Therefore, great efforts, such as CIPHER [4], MDGC [7], PageRank [9], DNRC [12], ToppGene [13], RWRH [14], MRF [15], and IBNPKATZ [16], have been devoted to the gene prioritization with an immediate purpose to improve the accuracy of prediction by exploring the topological structure of cancer networks. Compared with these classification-based methods, there are two advantages of network-based methods. First, the network-based algorithms do not require a large training set to rank genes. Second, these algorithms can explore the indirected relations among genes by exploiting the topological structure of networks, such as short paths and percolation. The difference among the network-based methods depends on how to make use of the topological structure of networks. For example, IBNPKATZ [16] prioritizes genes by combining the Katz index and network projection. RWRH [14] relies on the heterogeneous network structure, which adopts random walk to exploit gene-phenotype relationship. MRF [15] employs genes and subnetwork to explore gene-disease relation. PRINCE [32] adopts the information propagation of networks to rank genes, which precisely predicts disease-causing genes.

Even though network-based and similarity-based approaches have been successfully applied to gene prioritization, their performance is not desirable when the number of pathogenic genes is limited. Even worse, these algorithms are not applicable when the number of pathogenic genes is less than a threshold. However, the number of known pathogenic genes for many complex diseases, particularly for the rare diseases, is small because the current knowledge of them is limited. Recently, transfer learning [33–36] overcomes this problem by learning knowledge from source domains into the target domain with limited labelled objects, which significantly improves the performance of algorithms. More specifically, different from the traditional machine learning techniques, transfer learning aims to transfer knowledge from some previous tasks to a target task when the latter has a few of high-quality training data. It is also one of the major motivation of this study.

**Fig. 1:**
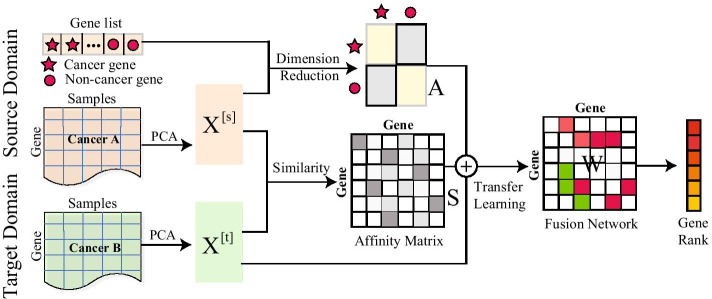
Overview of TLGP, which consists of the affinity matrix construction, dimension reduction in source domain, fusion network construction and gene ranking. Affinity matrix quantifies the similarity of genes between source and target domain. Dimension reduction learns the expression representation of source cancer, where cancer and non-cancer genes are well separated. The fusion network is based on the integration of source and target data. The gene ranking is performed via exploring fusion matrix

To improve the accuracy of gene ranking, we propose a novel transfer learning algorithm (called TLGP) for gene prioritization with few or even no pathogenic genes (called TLGP) in the target cancer, where transfers knowledge of cancers in source domains. The target cancer only compromises the gene expression profile, whereas the gene expression profiles and pathogenic genes of cancers exist in source domain. shown in Fig. 1, TLGP consists of four components: affinity matrix construction, dimension reduction in source domain, fusion network construction, and gene prioritization on the fusion network. Specifically, TLGP construct the affinity matrix quantifies the similarity of genes among various cancers. And, to obtain knowledge in cancers, we employ the dimension reduction to learn the low-dimensional representation of genes in the source cancers, where pathogenic and non-pathogenic genes are well separated. Then, TLGP automatically transfers knowledge from source domain into the target cancer and learns the gene similarity network for the target cancer, which is more reliable than that based on the gene expression profile of the target cancer. Finally, we prioritize genes in target cancer using a typical gene ranking algorithm.

In summary, the contributions of this study can be summarized as follows.

- A novel transfer learning algorithm for gene ranking is proposed, where the knowledge from other cancers can be transferred to the target cancer to improve the accuracy of algorithms. The TLGP algorithm also offers an alternative for integrative analysis of the heterogeneous genomic data.
- The proposed algorithm extends the application of algorithms for gene prioritization because it works well on cancers with no or limited pathogenic genes. It also serves as a flexible framework for gene prioritization.
- The experimental results demonstrate the proposed algorithm significantly improves the accuracy of algorithms.

**Fig. 2:**
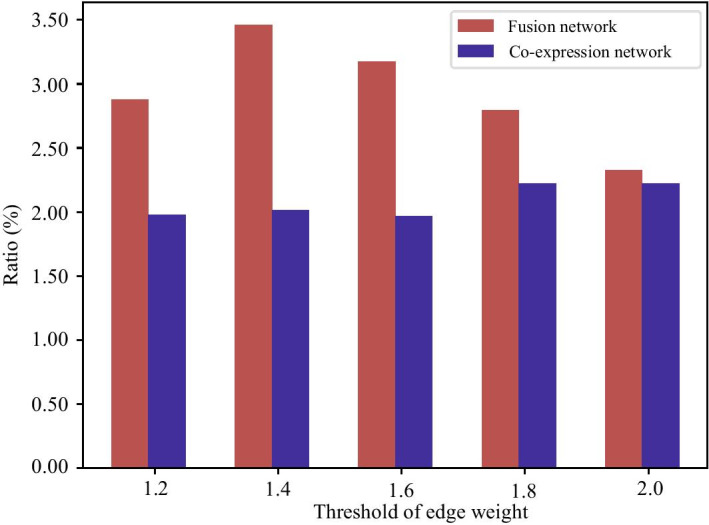
Percentages of edges in networks overlap with the biological experiment validated protein interactions on various values of parameter $\alpha$ from 1.2 to 2.0 with a gap 0.02, where red denotes the percentage of the fusion network and blue for the co-expression network

## Results and discussion

A comparative comparison is performed to fully validate the performance of the proposed algorithm.

**Fig. 3:**
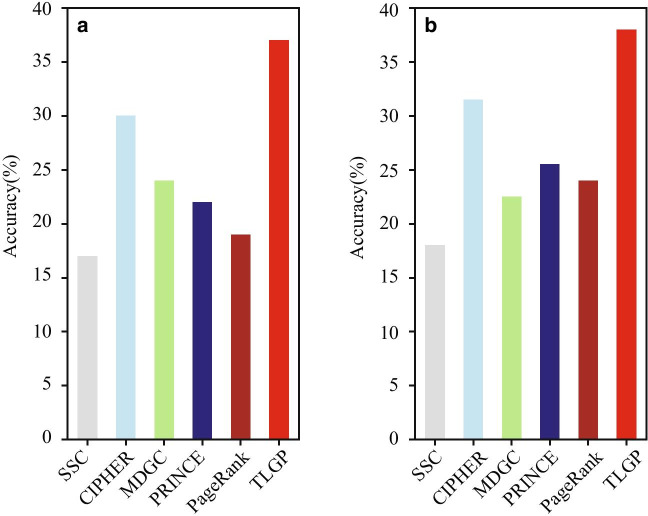
Performance of various algorithms on the pathogenic gene ranking, where the accuracy is the percentage of top *k* genes overlapped with the known pathogenic genes: **a** $k=100$, and **b** $k=200$

### Data and setting

We select breast and lung cancers as target and source domains, respectively. The pathogenic and non-pathogenic genes for breast and lung cancer are derived from COSMIC.^1^ The RNA-seq expression profiles of breast and lung cancer are downloaded from TCGA, where FPKM (Fragments Per Kilobase of transcript per Million fragments mapped) is used. The protein interaction network is downloaded from BioGRID.^2^ The pathogenic gene list for the breast cancer is used as benchmark to testify the accuracy of algorithms.

To fully validate the performance of the proposed algorithm on the gene prioritization, six state-of-the-art approaches, such as SSC [30], CIPHER [4], PRINCE [32], MDGC [7] and PageRank [9], are selected for a comparative comparison. These algorithms are selected because they achieve an excellent performance on the gene prioritization by using various strategy to exploit the topological structure of networks. For example, SSC [30], defines the similarity on the protein interaction network and use random walk on global network to detect disease-related genes, while CIPHER [4] constructs a regression model under the assumption that two closer genes in the molecular interaction network tend to cause similar phenotypes. SSC and CIPHER only explore the local information of networks to prioritize genes, while PRINCE [30] and PageRank [9] rank genes by using the random walk to explore the global information of networks with the underlying assumption that genes that cause similar diseases tend to be closed in the protein interaction network. MDGC [7] is a multi-view clustering method which generalizes the single-view discriminative K-means, and then prioritizes genes by making use of the degree of known diseases genes and statistical methods. All these algorithms run on the protein interaction networks to rank genes with the default values of parameters.

To measure the accuracy of algorithms, we check the number of pathogenic genes among the top *k* genes.

**Fig. 4:**
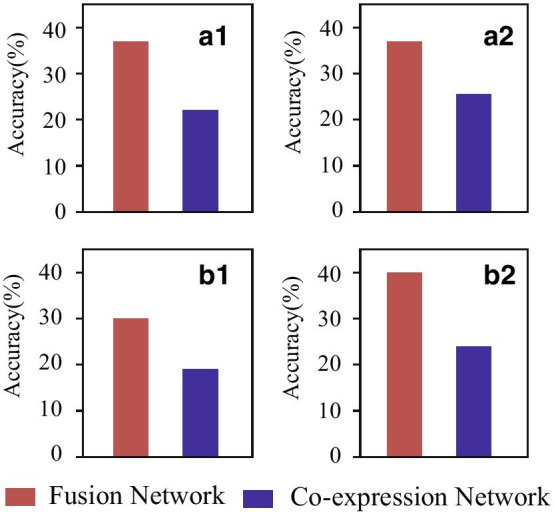
Accuracy of various algorithms for gene prioritization on the fusion and co-expression networks: **a1** PRINCE on top 100 genes, **a2** PRINCE on top 200 genes, **b1** PageRank on top 100 genes, and **b2** PageRank on top 200 genes, where red is for fusion matrix and blue for gene co-expression network

### Fusion network is more enriched by protein interactions

TLGP extracts knowledge in lung cancer and transfers it into breast cancer to construct the gene fusion network. Thus, it is natural to ask what is the difference between the learned fusion network and gene co-expression network based on the gene expression profiles, i.e., which one is better.

To address this issue, the biological experiment validated protein interactions are selected as the gold standard to measure the quality of the fusion network. We check the percentage of edges in the fusion and gene co-expression network that overlap with the protein interactions. Since both fusion and co-expression networks are weighted, we select these edges in each network whose weights are greater than a predefined threshold. The percentages of edges overlapping with the protein interactions for the fusion and co-expression networks on various thresholds are shown in Fig. 2. The threshold is defined as $\alpha \times$ mean of edge weights in network, where the red bar denotes the percentage of the fusion network constructed by TLGP and the blue represents that of the gene co-expression network. From Fig. 2, it is easy to assert that the edges in fusion network are more enriched by the protein interactions than the gene co-expression network at all thresholds. Specifically, 2.8% of edges in fusion network are overlapped with protein interactions, while only 1.9% for gene co-expression network when $\alpha$=1.2. These result indicates that the fusion network is more reliable than the gene co-expression network, implying that transferring knowledge from other cancers improves the accuracy of network construction. There are two possible reasons to explain why the fusion network constructed by TLGP is more reliable than the gene co-expression network. First, the integrative analysis of the gene expression and pathogenic gene list remove the noise in the source cancer. Second, the knowledge in the source cancer is transferred to the fusion networks, thereby improving the quality of the fusion network.

**Fig. 5:**
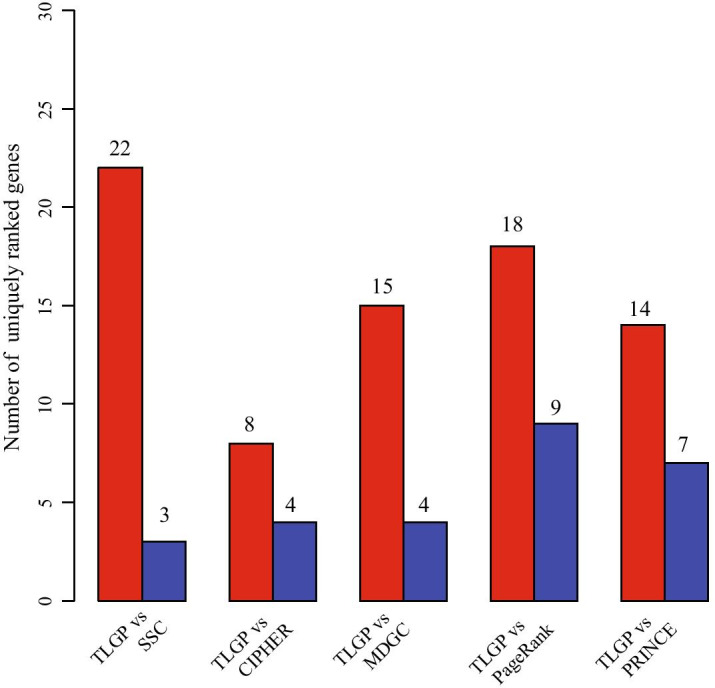
The number of uniquely ranked pathogenic genes among the top 100 genes obtained by the various algorithms

### Performance on ranking pathogenic genes

Figure 2 demonstrates the proposed algorithm can remove noise in genomic data and constructs the reliable fusion network. Then, we ask whether the constructed fusion network can improve the accuracy of gene prioritization. To comprehensively testify the performance of the proposed algorithm, we use two types of gene lists, such as pathogenic and cancer causal genes, to evaluate the performance of algorithms.

The percentage of top *k* genes that are overlapped with the known pathogenic genes is shown in Fig. 3, where panel a is the accuracy of various algorithms with *k*=100 and panel b with *k*=200. From Fig. 3a, it is easy to conclude that the accuracy of TLGP is significantly higher than the others. CIPHER are inferior to TLGP, and it is much more precise than the SSC, MDGC, and PRINCE. The SSC algorithm is the worst. The reason is that it only exploits the local topology of networks, which fails to characterize the centrality of genes in the networks. Specifically, the accuracy of TLGP is 38.0%, which is 7% higher than that of when the top 100 genes are selected. There two reasons to explain why TLGP significantly outperforms the others. First, TLGP integrates heterogeneous genomic data for gene prioritization, thereby providing a better strategy to characterize the centrality of cancer related genes. Second, TLGP transfers knowledge from the source cancer to the target cancer, which improves the reliability and accuracy of the fusion network. The comparison between TLGP and PRINCE further demonstrates that the transfer learning strategy can significantly improve the accuracy of gene prioritization. Figure 3b shows the accuracy of algorithms on gene prioritization with *k*=200, where the similar tendency repeats.

**Fig. 6:**
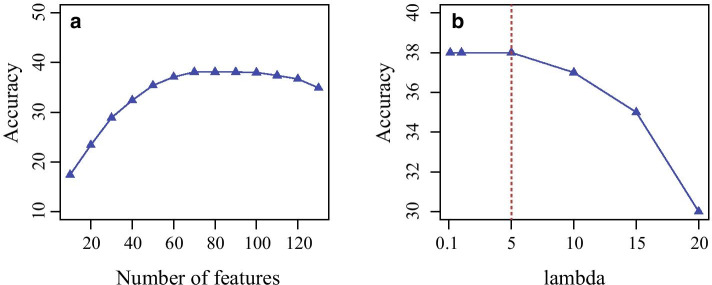
Parameter effect: **a** how the number of features effects the performance of TLGP, and **b** how parameter $\lambda$ effects the accuracy of TLGP with various values

The proposed algorithm adopts PRINCE for gene prioritization. Then, we ask whether the excellent performance of TLGP is co-factor by the PRINCE algorithm [32]. We apply two algorithms, such as PRINCE [32] and PageRank [9], on the fusion and gene co-expression networks. The results are presented in Fig. 4, where panel a1 and a2 contain the accuracy of PRINCE on these two types of networks, and panel b1 and b2 are those of PageRank. It is easy to conclude that all these algorithms achieve a much better performance on the fusion network than that on the gene co-expression network. These results imply the superiority of the proposed algorithm on gene prioritization.

The above experiment validate the percentage of top *k* genes overlapped with the pathogenic genes, which is insufficient to fully validate the performance of algorithms for gene prioritization. Here, we investigate the uniquely identified pathogenic genes, i.e., these pathogenic genes that only can be discovered by a specific genes in the top *k* genes. To make a comprehensive comparison, we compare TLGP with the others to investigate whether the proposed method is efficient to rank the pathogenic genes that cannot be obtained by others. The results are shown in Fig. 5, where the red bar denotes the number of uniquely ranked genes by using TLGP and the blue one represents that of the others. From our proposed algorithm TLGP achieves the best results when compares with SSC, PageRank, PRINCE, MDGC and CIPHER. From Fig. 5, we assert that the proposed algorithm can identify much more pathogenic genes than the others. For example, there are 22 uniquely ranked genes among the top 100 genes obtained by TLGP, whereas there are 3 uniquely ranked genes by SSC. Compared TLGP with CIPHER, MDGC, PRINCE, and PageRank, there are 8, 15, 18, 14 uniquely ranked genes in top 100 genes obtained by TLGP, and those are 4, 4, 9, 7, respectively. These results further demonstrate the proposed algorithm can identify the pathogenic genes of the breast cancer that cannot be discovered by the other algorithms, indicating the superiority of TLGP for gene prioritization. The possible reason is that the functions of some pathogenic genes are complex that cannot be fully characterized by using one type of genomic data. TLGP integrates the heterogeneous genomic data, improving the accuracy of prediction.

### Parameter sensitivity

Finally, we investigate how the parameters effect the performance of the proposed algorithm. Notice that two parameters are involved, where the number of features for dimension reduction, and parameter $\lambda$ determines the importance of penalty. TLGP empirically select the best values for parameters.

Specifically, TLGP requires the gene expression profiles in the source and target cancer have the same numbers of features. When the dimensions of the gene expression profiles are not consistent, TLGP makes use of Principal Component Analysis (PCA) to project the gene expression profiles into a space where the numbers of features are the same. How the accuracy of TLGP changes as the number of features increases from 10 to 130 with a gap 10 is shown Fig. 6a. The accuracy of TLGP improves as the number of features increases from 10 to 70, whereas the performance of the proposed algorithm declines as the number of features increases from 100 to 130. And, the accuracy is stable when the number of features is in [70,100]. When the number of features is small, these features are insufficient to fully characterize the information of gene expression data, thereby resulting in the low accuracy. When the number of features is large, the features are redundant, thereby leading to the decrease of accuracy. When the number of features is in [70,100], TLGP achieves a good balance. Thus, we set the number of features as 80.

Then, we investigate how the parameter $\lambda$ for the penalty effects the performance of TLGP. How the accuracy of the proposed algorithm changes as $\lambda$ increases from 0.01 to 15 is shown in Fig. 6b. The performance of TLGP achieves the best performance when $\lambda \in$[0.01, 5]. The accuracy of TLGP decreases as parameter $\lambda$ increases from 5 to 15. The reason is that when the value of *lambda* is large, the penalty dominates the objective, resulting in the low accuracy. In this study, we set *lambda*=1.

## Conclusions

Gene ranking is one of the fundamental problems in bio-informatics, which are critical for the cancer diagnosis and therapy. The existing algorithms make use of the networks and cancer-causing genes to predict the centrality of genes. However, these algorithms are criticized for their low accuracy when the number of cancer-causing genes is limited. Furthermore, these algorithms cannot be applied to the gene prioritization when no known cancer-causing gene is available. Actually, the number of cancer-causing genes for many cancers is limited, particularly for these rare diseases. To solve this problem, we propose a transfer learning based algorithm for gene prioritization with no pathogenic genes in target cancer, where knowledge in the source cancers is incorporated into the target cancer to improve the performance of algorithms. The experimental results demonstrate that the proposed algorithm significantly outperforms the current algorithms on the gene ranking.

The proposed algorithm also has some limitations, which will be improved by further research:

- The gene expression profiles in the source and target cancers have the same distributions because they are generated by using the platform. How to transfer knowledge for the heterogeneous genomic data from the source domain to target domain, such as the gene expression in the source domain and methylation data in the target domain, is also promising to further improve the performance of gene ranking.
- In this study, only one source cancer is adopted for transfer learning. How transfer knowledge from the multiple source domains is also a critical problem for gene prioritization.

Designing effective and efficient algorithms to address the above two issues would be promising for gene prioritization.

## Methods

In this section, we address the objective function, optimization and analysis of algorithms are successively addressed.

### Preliminaries

Before describing the details of TLGP, let us introduce some notations that are widely used in the next subsections.

In this study, matrices are denoted by capital letters, and vectors by bold lowercase letters. Given the gene expression profiles as an matrix *X* with the *i*th row and *j*th column element $x_{ij}$, where the row denotes a gene and the column corresponds to a patient. The *i*th row (column) is denoted by $\mathbf{x} _{i.} (\mathbf{x} _{.j})$. $X^{'}$ is the transpose of *X*. Let $X^{[s]} \in R^{n\times d^{[s]}}$ and $X^{[t]} \in R^{n\times d^{[t]}}$ be the gene expression profiles of the source and target cancer, respectively. Let the binary vector $\mathbf{y} =\{y_{1},\ldots ,y_{n}\}$ is an indicator for the pathogenic genes in the source cancer, where $y_{i}$=1 if the *i*th gene is pathogenic, 0 otherwise.

Given an undirected and weighted network $G=(V,E)$ with vertex set $V=(v_{1},\ldots ,v_{n})$ (*n* is the number of node) and edge set $E=\{(v_{i},v_{j})\}$, the weighted adjacent matrix $W=(w_{ij})_{n\times n}$ is constructed, where element $w_{ij}$ denotes the weight on edge $(v_{i},v_{j})$. If *G* is an un-weighted network, $w_{ij}$ is 1 if $v_{i}$ and $v_{j}$ are connected, 0 otherwise. Let $w_{i.}(w_{.j})$ be the *i*th row (*j*th column) of *W*. All networks are undirected, i.e. $W^{'}=W$. The degree of the *i*th node is defined as the sum of weights on edges connecting to vertex $v_{i}$, i.e., $d_{i}=\sum _{j}w_{ij}$. The degree matrix is the diagonal of degree sequence, i.e. $D=diag(d_{1},\ldots ,d_{n})$, and the Laplacian matrix of *W* is defined as $L_{W}=D-W$. Given a network $G=(V,E)$, a similarity matrix *S* can be constructed, where element $s_{ij}$ denotes the similarity between vertex $v_{i}$ and $v_{j}$. The gene prioritization in a network $G=(V,E)$ is to construct a function $\psi$ to measure the centrality of vertices, i.e.,

1 $$\begin{aligned} \psi : V\mapsto \mathcal {R}^{+}, \end{aligned}$$

where $\mathcal {R}^{+}$ denotes the interval $(0,+\infty )$.

### Objective function

The overview of the proposed algorithm is shown in Fig. 1, which consists of the affinity matrix construction, dimension reduction in source domain, fusion network construction and gene ranking. The ultimate goal of TLGP is to learn a reliable and fused network for genes, where the heterogeneous genomic data from the source and target domains are integrated by using transfer learning. In transfer learning, two critical techniques are involved, i.e., how to extract knowledge from source domain and how to transfer knowledge to target domain, which are also two factors for the objective function of the proposed algorithm.

To transfer knowledge in the source cancer, we need to quantify the similarity between the source and target cancer because it decides where the knowledge can be extracted. The purpose of domain adaptation is to use labeled data in the source domain to improve the performance of the target task when the target domain is similar to the source domain. However, when the distributions of the source and target domain differ greatly, the performance of transfer learning is undesirable. To solve this problem, many methods [37–40] explore how to narrow the difference in the distribution of features between the two domains through some transformations. For example, TCA [37] assumes that the marginal distribution between source domain and target domain is different but there exist a mapping function $\Phi (.)$ that projects two domains into a common space in which the discrepancy will be minimized. JDA [38] considers that both marginal distribution and conditional distribution between source domain and target domain are different and proposes to iteratively use the pseudo labels to approximate the true labels.

In this study, the distributions of the source and target cancer differ greatly because the gene expression profile and pathogenic genes are involved in the source cancer, whereas the target cancer only has the expression data. Therefore, we need to integrate the gene expression and pathogenic gene list. However, it is difficult to integrate the genomic data, particularly for the heterogeneous data [41]. To solve this problem, we use the pathogenic gene list to adjust the gene expression profiles with the underlying assumption that the pathogenic and non-pathogenic genes have different expression patterns. Thus, we expect to learn a representation for $X^{[s]}$, denoted by *A*, such that the expression profiles of pathogenic and non-pathogenic genes are well separated, which can improve the accuracy of algorithms. LMNN [42] is adopted for this issue, which obtains new representation of the gene expression profiles of the source cancer using a project matrix $H^{[s]}\in R^{k\times r}$ by minimizing the approximation between the expression data and representation, i.e.,

2 $$\begin{aligned} \min \Vert A-X^{[s]}H^{[s]}\Vert ^{2} \end{aligned}$$

where $A\in R^{n\times r}$ is the new representation of $X^{[s]}$ .

Then, we consider how to transfer learning between the source and target cancer based on the gene expression profiles by constructing the affinity matrix $S\in R^{n\times n}$, element $s_{ij}$ denotes the absolute value of Pearson coefficient between $\mathbf{x} _{i.}^{[s]}$ and $\mathbf{x} _{j.}^{[t]}$. The underlying assumption is that genes with the same or similar functions have the same or similar expression patterns. Thus, if a pair of genes have the similar expression patterns in the source and target cancer, we have enough reasons to believe that they share knowledge. If the *i*th gene in target cancer is similar to the *j*th gene in the source genes in terms of gene expression, we can transfer the knowledge between them. One issue that must be solved before transferring knowledge is to quantify how similarity they because it determines how much information can be transferred. The expression profile of the *i*th gene must be consistent with the representation in Eq. (2). We learn a project matrix *S* to measure the distance between them, i.e.,

3 $$\begin{aligned} \Vert \mathbf{x} _{i.}^{[t]}U-\mathbf{a} _{j.}\Vert ^{2}, \end{aligned}$$

where $\mathbf{a} _{j.}$ is the *j*th row in *A*, and $\Vert A\Vert$ is the Frobeneous norm of *A*. However, Eq. (3) quantifies the similarity in terms of gene expression profiles, ignoring the similarity of genes *S*. Actually, the shared knowledge for transferring is also determined by the similarity of gene pair. Thus, we weight the distance in Eq. (3) by using the similarity matrix *S*, which is re-written as

4 $$\begin{aligned} s_{ij}\Vert \mathbf{x} _{i.}^{[t]}U-\mathbf{a} _{j.}\Vert ^{2}. \end{aligned}$$

Analogously, we expect in the fused network $w_{ij}$ receives heavy weight if the corresponding gene pair have the similar expression profiles in target domain, i.e.,

5 $$\begin{aligned} w_{ij}\Vert (\mathbf{x} _{i.}^{[t]}-\mathbf{x} _{j.}^{[t]})U\Vert ^{2}. \end{aligned}$$

By combining Eqs. (4, 5), we obtain the objective function as

6 $$\begin{aligned} \frac{1}{2}\sum _{i,j}(s_{i,j}\Vert \mathbf{x} _{i}^{[t]}U-\mathbf{a} _{j}\Vert ^{2} +w_{ij}\Vert (\mathbf{x} _{i}^{[t]}-\mathbf{x} _{j}^{[t]})U\Vert ^{2}+\lambda \Phi (w_{i,j})), \end{aligned}$$

where $\Phi (w_{i,j})$ is a penalty item, and parameter $\lambda$ controls the importance of the penalty item (how parameter $\lambda$ effects the performance is investigated in the experiments). The criterion for $\Phi (w_{i,j})$ is that it is close to 0 when there exist an strong connection between the *i*th and *j*th genes, 1 otherwise. Here, we set it as $(\sqrt{w_{i,j}}-1)^{2}$.

In the next subsection, we deduce the optimization rules for the minimization problem in Eq. (6).

### Optimization

Equation (6) involves two variables *U* and *W* because the matrix *A* is learned by using LMNN [42]. However, it is difficult to directly optimize Eq. (6) because of the non-convexity. An iteration strategy is employed to optimize Eq. (6), where one variable is updated by fixing the other. The iteration continues until the algorithm is convergent.

Fixing *U*, we obtain the update rule for $w_{i,j}$ as

7 $$\begin{aligned} w_{i,j}=\left(\frac{\lambda }{\Vert (\mathbf{x} _{i}^{[t]}-\mathbf{x} _{j}^{[t]})U\Vert ^{2}+\lambda }\right)^{2} \end{aligned}$$

When *W* is fixed, the second item of the objective function can be formulated as

8 $$\begin{aligned} \sum _{i,j}w_{i,j}\Vert (\mathbf{x} _{i}^{[t]}-\mathbf{x} _{j}^{[t]})U\Vert ^{2}=tr(L_{W}X^{[t]}UU^{'}(X^{[t]})^{'}), \end{aligned}$$

where $L_{W}$ is the Laplacian matrix of *W*. Furthermore, the first item of Eq. (6) can also be transformed into matrix trace as

9 $$\begin{aligned} tr(DX^{[t]}UU^{'}(X^{[t]})^{'})-2tr(SX^{[t]}UA^{'})+tr(DAA^{'}), \end{aligned}$$

where *D* refers to the degree matrix of *S*.

Submitting Eqs. (8) and (9), the objective function is written as

10 $$\begin{aligned} \begin{aligned} \Theta&=\frac{1}{2}(tr(DX^{[t]}UU^{'}(X^{[t]})^{'})\\&\quad -2tr(SX^{[t]}UA^{'})+tr(DAA^{'})\\&\quad +tr(L_{W}X^{[t]}UU^{'}(X^{[t]})^{'})+\sum _{i,j}\lambda \Phi (w_{i,j})) \end{aligned} \end{aligned}$$

The the partial derivative of *U* is deduced as

11 $$\begin{aligned} \frac{\partial {\Theta }}{\partial {U}}=(X^{[t]})^{'}LX^{[t]}U+(X^{[t]})^{'}DX^{[t]}U+(X^{[t]})^{'}SA. \end{aligned}$$

According to KKT condition, by setting $\frac{\partial {\Theta }}{\partial {U}}$=0, we obtain the update rule for *U* as

12 $$\begin{aligned} U=U-\alpha ((X^{[t]})^{'}LX^{[t]}U+(X^{[t]})^{'}DX^{[t]}U+(X^{[t]})^{'}SA). \end{aligned}$$

After obtain the fused network *W*, typical algorithms for gene prioritization, such as PRINCE [32], to rank genes in the target cancer. The procedure of TFGP is presented in Algorithm 1.

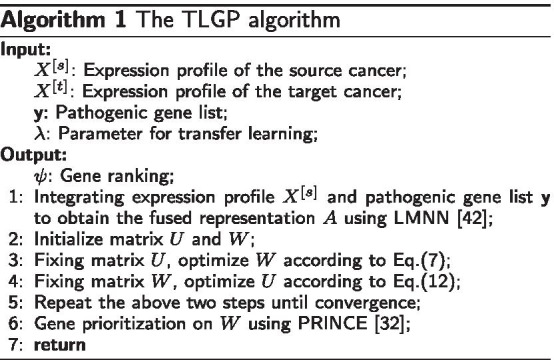



### Algorithm analysis

On the space complexity, the expression profile of source and target domain requires space *O*(*nm*), where *m* is the maximum of the numbers of samples in the source and target cancer, i.e., $m = ma,x\{d^{[s]} , d^{[t]}\}$. The fusion matrix *W* and similarity matrix *S* requires space $O(n^{2})$. Therefore, the overall space complexity is $O(n^{2}+nm)=O(n^{2})$ because $m\ll n$, demonstrating that the proposed method is efficient in terms of the space complexity.

On the time complexity, the time for update *W* is $O(n^{2})$. The running time for updating *U* is $O(n^{2}m)$. Thus, the total running time is $O(l(n^{2}+n^{2}m)=O(n^{2}lm)$, where *l* is the number of iterations. It is the same as that of nonnegative matrix factorization [43].

## Data Availability

The data are publicly available in TCGA (https://portal.gdc.cancer.gov/), and COSMIC (https://cancer.sanger.ac.uk/cosmic/).

## Abbreviations

SVM: Support vector machine
TCGA: The Cancer Genome Atlas
FPKM: Fragments Per Kilobase of transcript per Million fragments mapped
COSMIC: Catalogue Of Somatic Mutations In Cancer
NMF: Nonnegative matrix factorization

## Footnotes

1 https://cancer.sanger.ac.uk/cosmic/.

2 https://thebiogrid.org/.
